# Progressive Spatiotemporal Graph Modeling for Spacecraft Anomaly Detection

**DOI:** 10.3390/e28040426

**Published:** 2026-04-10

**Authors:** Zihan Chen, Zewen Li, Yuge Cao, Yue Wang, Hsi Chang

**Affiliations:** 1 School of Advanced Manufacturing and Robotics, Peking University, Beijing 100871, China; 2 Institute of Remote Sensing Satellite, China Academy of Space Technology, Beijing 100094, China

**Keywords:** spacecraft anomaly detection, satellite telemetry data mining, spatial–temporal graph, Explainable AI (XAI) for aerospace

## Abstract

The growing number of on-orbit spacecraft and the increasing volume of telemetry data have made intelligent anomaly detection in multi-channel telemetry essential for mission operations. Current spacecraft anomaly detection methods primarily rely on statistical models or time-series deep learning approaches, which often fail to explicitly model spatiotemporal dependencies across multiple telemetry channels. This shortcoming limits their ability to capture the dynamically evolving and intricately coupled relationships between variables. To overcome this limitation, a Progressive Spatiotemporal Graph (PSTG) model is proposed for anomaly detection in multi-channel spacecraft telemetry. PSTG employs a multi-scale patch embedding module to extract hierarchical semantic features from multi-channel time series, effectively reducing the dimensionality of the spatiotemporal graph. It constructs a sparse adjacency matrix using a multi-head attention mechanism that integrates intra-channel temporal dynamics, inter-channel spatial correlations, and cross-channel spatiotemporal interactions. An improved multi-head graph attention network then captures pairwise dependencies among nodes within the adjacency matrix. As a result, PSTG encodes rich spatiotemporal representations derived from intricate variable interactions, enabling accurate, real-time prediction of multi-channel telemetry. Furthermore, a dynamic thresholding mechanism is incorporated into PSTG to perform online anomaly detection based on prediction residuals. Extensive experiments on real-world spacecraft telemetry data collected over 84 months show that PSTG outperforms eleven state-of-the-art benchmark methods in almost all cases across multiple evaluation metrics. Finally, visualizations of the learned adjacency and attention matrices are presented to interpret the spatiotemporal modeling process, providing operators with actionable insights into the detected anomalies and facilitating root cause analysis.

## 1. Introduction

Satellites are sophisticated systems composed of multiple components, each serving distinct functions. Due to the extreme operational environments, such as rapid thermal cycling and intense electromagnetic radiation, it is difficult to prevent operational anomalies and failures, which pose significant risks to in-orbit satellite reliability and safety [1]. To mitigate these risks, satellite operators typically monitor key time-series telemetry data continuously, aiming to detect anomalies early and prevent critical system failures that could disrupt mission operations [2]. However, modern satellites generate vast amounts of telemetry data, including parameters such as temperature, voltage, and current [3]. Manually inspecting such high-volume, multivariate data for anomalies is highly labor-intensive, requiring operators to track hundreds of interrelated parameters across various subsystems. Although feature dimensionality reduction techniques can partially alleviate this burden, prior research indicates their limited effectiveness when applied to large-scale, high-dimensional sequential data [4]. In response, this study directly focuses on modeling multidimensional telemetry time series to preserve cross-parameter correlations and enhance anomaly detection accuracy. Given the complex interdependencies among telemetry variables, addressing anomaly detection within a multivariate time series framework is essential for reliably identifying significant deviations [4].

Telemetry data exhibits high dimensionality and dynamic interaction patterns over time. These interactions manifest as temporal, spatial, and spatiotemporal correlations across multiple channels. Temporal correlation refers to the dependence of current values on historical observations within a single channel, driven by periodic behaviors and underlying system dynamics. Spatial correlation arises from physical and functional dependencies among different subsystems, where the state of one telemetry channel influences others at the same moment. Furthermore, due to causal relationships (e.g., an increase in motor speed leading to delayed rises in current and temperature), telemetry data is also affected by past information from other channels, reflecting broader spatiotemporal dependencies. These non-exclusive correlation types may coexist and evolve dynamically throughout a mission. Beyond easily identifiable point anomalies, contextual anomalies, i.e., values that appear normal in isolation but deviate under specific temporal or operational conditions [1,5,6], require a deep understanding of the intricate spatiotemporal structure of multichannel telemetry. Consequently, spacecraft anomaly detection remains heavily reliant on expert analysts, and accurate, intelligent, and automated detection continues to pose a major challenge.

Early efforts in spacecraft anomaly detection were pioneered by NASA and its affiliated research centers. Systems such as the Inductive Monitoring System (IMS) [7], the BEAM/DIAD framework [8], and related approaches [9,10] laid the foundation for rule-based and data-driven health monitoring of shuttle telemetry, employing clustering-based nominal modeling and statistical invariants to identify deviations from normal behavior. With the rapid development of deep learning, substantial progress has been made in detecting anomalies in multivariate time series. Recurrent Neural Networks (RNNs) [11] and Long Short-Term Memory (LSTM) networks [12] excel at capturing temporal dependencies, while Convolutional Neural Networks (CNNs) [13], Variational Autoencoders (VAEs) [14], and Graph Neural Networks (GNNs) [15] are used to model inter-variable relationships. However, most of these models implicitly encode spatiotemporal interactions within global hidden states, failing to explicitly represent the underlying dependency structures. Spatiotemporal Graph Neural Networks (STGNNs) [16] offer a more structured approach by using GNN modules [17] to model spatial correlations and CNN [18], LSTM [19], or Transformer [20] components to capture temporal dynamics. Despite these advances, existing methods often fail to jointly model evolving spatiotemporal dependencies across channels and time steps. Temporal modeling is typically confined to individual channels, and spatial relationships are encoded without temporal context, resulting in poor representations of cross-channel phenomena such as delayed responses or coupled oscillations. This limitation undermines the detection of correlated or system-level anomalies.

To address these challenges, a prediction-driven anomaly detection framework is adopted, which first forecasts future telemetry values and then identifies anomalies based on the prediction residuals. The primary contribution of this work lies in the tailored design of a novel and robust forecasting model referred to as Progressive Spatiotemporal Graph (PSTG).

The main contributions of this paper are summarized as follows:

(a) A novel multi-scale adaptive fusion method is proposed to address the challenge of simultaneously capturing global patterns and local variations in spacecraft telemetry across diverse mission profiles. By modeling both long-term dependencies and short-term fluctuations, the method enables a comprehensive temporal feature representation that surpasses the capabilities of conventional single-scale approaches.

(b) A unified spatiotemporal graph representation, enhanced with an adaptive attention mechanism, is introduced to overcome the limitations of static dependency modeling. This approach dynamically identifies the most relevant node interactions at each time step, enabling the simultaneous learning of heterogeneous spatiotemporal dependencies through a single coherent graph structure, thereby significantly improving the modeling accuracy of complex spacecraft systems.

(c) The effectiveness of the complete PSTG framework is demonstrated through extensive experiments on a real-world spacecraft telemetry dataset spanning 84 months. While the model outperforms eleven state-of-the-art methods in almost all cases across multiple metrics, more importantly, its learned graph structure supports interpretable analysis. This capability is critical for assisting operators in diagnosing the root causes of detected anomalies.

The remainder of this paper is organized as follows. Section 2 reviews related work. Section 3 presents the architecture and technical details of the PSTG framework. Section 4 evaluates the proposed method on a real-world spacecraft telemetry dataset. Section 5 concludes the paper.

## 2. Related Work

### 2.1. Foundational Spacecraft Anomaly Detection Methodologies

Early spacecraft anomaly detection techniques primarily relied on threshold-based rules and expert systems. Threshold methods involve setting fixed upper and lower bounds for each telemetry channel, making them ineffective for detecting contextual or collective anomalies [21]. Expert system-based approaches [22] require manually defined rules and are typically limited to monitoring only a few critical subsystems. These methods struggle to detect previously unseen anomalies or novel fault signatures, resulting in high false-negative rates, particularly in advanced spacecraft used for deep space missions.

With advancements in digital modeling, model-based anomaly detection has gained traction. These methods rely on constructing an accurate digital model of spacecraft systems, comparing simulated outputs with actual telemetry, and identifying discrepancies as potential anomalies. Kolcio et al. [23] developed a nominal model of a spacecraft’s attitude control system using Simulink and SysML, applying the constraint suspension method for fault diagnosis. However, as spacecraft become more autonomous and complex, creating precise digital models becomes increasingly challenging. Difficulties in parameter back-solving and real-time model updating severely limit the practicality and scalability of such approaches.

The growing volume of telemetry data and advances in artificial intelligence have spurred interest in data-driven anomaly detection. These methods leverage raw time series data and machine learning algorithms to distinguish between normal and anomalous states without requiring explicit fault rules. In recent years, classification-, clustering-, and prediction-based machine learning techniques have emerged as prominent tools for spacecraft anomaly detection.

Classification-based methods typically employ supervised learning, which can be either statistical or distance-based. Bernal-Mencia et al. [24] used Kernel Principal Component Analysis (KPCA) for feature extraction, followed by a Multi-Layer Perceptron (MLP) for binary classification of normal and abnormal states. However, supervised methods demand large volumes of labeled training data, which are expensive and time-consuming to obtain, especially given the scarcity of documented anomalies in real missions [25]. As a result, unsupervised approaches such as clustering have gained increasing attention [26]. For instance, Li et al. [27] used normal-state telemetry as a reference baseline, computed distance scores for incoming sub-sequences, and performed anomaly detection based on similarity measures. Nevertheless, classification and clustering methods often lack sensitivity to fine-grained temporal changes, making them prone to missing short-lived anomalies and limiting their real-time applicability.

Prediction-based anomaly detection methods use algorithms, particularly deep learning models, to learn the normal behavior of telemetry data during regular operation. These models reconstruct historical data or predict future values under normal conditions [28], and anomalies are identified by analyzing deviations between predicted and observed values. Commonly used models include Trajectory Optimization [29], Linear Models [30], Relevance Vector Machines (RVMs) [31], Extreme Learning Machines (ELMs) [32], Autoencoders (AEs) [33], Bayesian Neural Networks (BNNs) [34], Temporal Convolutional Networks (TCNs) [35], RNNs [36], LSTMs [6,37], Transformers [14], and Generative Adversarial Networks (GANs) [4]. The LSTM-based Telemanom framework, developed by NASA engineers [6], has become a benchmark in satellite telemetry anomaly detection. Lakey et al. [38] compared various deep learning architectures and found CNNs effective for diagnosing multiple fault types, while LSTMs and RNNs excelled in capturing time-series anomalies. Transformers demonstrated strong performance in detecting subtle, prolonged anomalies but required substantial computational resources.

Critically, while deep learning models like LSTMs and early Transformers have significantly improved detection accuracy, the technical landscape has been further enriched by advanced architectures such as iTransformer [39], PatchTST [40], and efficient mixing structures like TSMixer [41] and WPMixer [42]. Despite these breakthroughs—including cross-dimensional dependency modeling [43], frequency-domain MLPs [44], and selective representation spaces [45]—a fundamental limitation persists: these models predominantly operate as “black boxes”. Although they can flag anomalies with high precision, they rarely provide interpretable explanations regarding why an anomaly occurred or which specific sensor interactions contributed to the detection. Furthermore, as Zeng et al. [46] highlighted, the inherent complexity of Transformer-based architectures does not always translate to superior performance in time-series tasks, potentially obscuring the decision-making process. This lack of transparency poses a significant barrier in safety-critical aerospace applications, where trust, accountability, and rigorous root-cause analysis are paramount. Consequently, the research frontier is shifting from pure accuracy enhancement toward the development of explainable anomaly detection frameworks that provide actionable insights for mission operations.

Beyond spacecraft-specific anomaly studies, recent fault-diagnosis research has also shown that unsupervised and partial domain adaptation strategies can improve robustness under distribution shift and class mismatch [47,48]. This trend is relevant to telemetry anomaly detection, where cross-mission transfer and rare-event imbalance remain practical challenges.

### 2.2. Spacecraft Anomaly Detection with Explainability

In spacecraft operations, it is not sufficient to merely detect anomalies with high accuracy; ground operators must also understand the reasons behind each detection. Clear explanations enable faster decision-making, reduce mission risk, and support corrective actions. Therefore, Explainable Artificial Intelligence (XAI) [49,50] has become essential in this field, providing transparent justifications for anomaly detection decisions. Hundman et al. [6] employed an LSTM model to predict single-channel telemetry and introduced a dynamic threshold mechanism for anomaly detection. This approach offers partial explainability and has been successfully deployed for the Soil Moisture Active Passive (SMAP) satellite and the Mars Science Laboratory (MSL) rover. However, it treats multichannel telemetry as independent sequences, neglecting inter-sensor dependencies. As a result, it cannot distinguish whether a prediction error stems from genuine system anomalies or modeling inaccuracies.

To address these limitations, Xu et al. [51] integrated attention mechanisms with LSTM to jointly learn inter-parameter correlations and long-term temporal dependencies, using dynamic thresholds for final detection. Yu et al. [52] utilized GNNs to model intrinsic properties of telemetry variables and applied attention mechanisms to capture short-term cross-dimensional interactions, followed by LSTM-based temporal feature extraction. Liu et al. [18] employed dynamic graph attention networks to model complex spatial correlations in multivariate time series and applied optimal thresholds for anomaly detection. Zeng et al. [12] constructed a feature attention-based LSTM and causal network to infer causal relationships among telemetry parameters, updating dynamic thresholds via the k-sigma method.

While these studies incorporate aspects of spatial or temporal modeling, they typically treat these dimensions separately, failing to fully integrate spatiotemporal dynamics. Some works attempt joint modeling: Tian et al. [19] used a graph attention network to capture spatial dependencies and an LSTM for temporal modeling, leveraging spatiotemporal information for anomaly detection. Yu et al. [13] combined the CNN and LSTM to extract spatiotemporal features and used GAN-generated anomaly scores to detect multichannel anomalies. However, since these approaches consider the temporal and spatial features separately, they fail to further analyze the explainable patterns, such as the temporal, spatial, and spatiotemporal correlations [53], in the time series data. Given the complex spatiotemporal dynamics of multi-channel spacecraft telemetry data, anomaly detection methods must account for these spatiotemporal characteristics in a unified manner.

## 3. Methodology

**Engineering-level intuition of PSTG:** Before presenting the formal mathematical derivations, this section first summarizes the practical operation logic of PSTG for spacecraft health monitoring. The framework continuously processes incoming telemetry in three tightly coupled steps: it first uses multi-scale patches to capture both short-term high-frequency transients and long-term low-frequency mission drifts; it then performs dynamic graph construction and structure-guided weighting to discover data-adaptive sensor couplings rather than relying on a fixed engineering schematic; finally, it compares predicted normal behavior with real-time observations and converts residual deviations into actionable alarms through a dynamically calibrated statistical threshold.

### 3.1. Overall Framework

**Figure 1:**
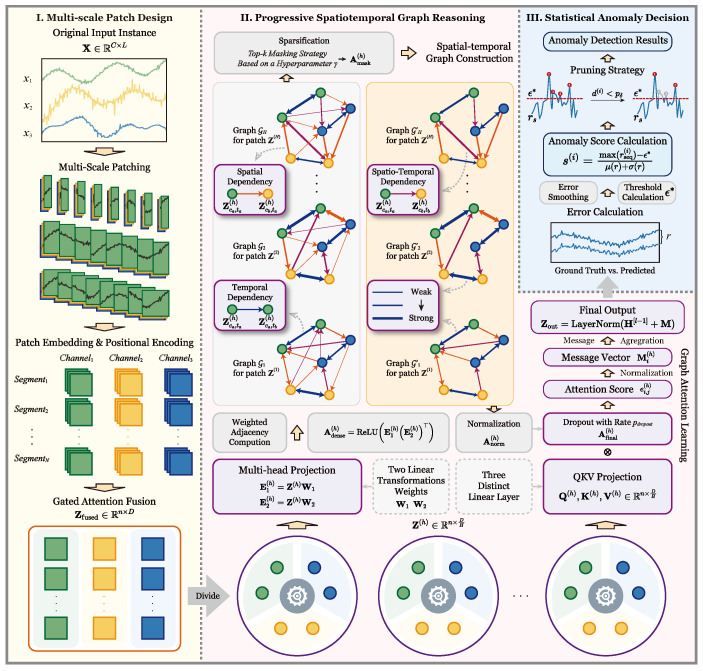
Overall framework of PSTG. The data flow proceeds through three key modules: (I) Multi-scale Patch Design: Raw inputs are segmented into multi-scale patches and mapped into high-dimensional embeddings via gated attention fusion. (II) Progressive Spatiotemporal Graph Reasoning: The module employs a sequence of graph attention blocks to repeatedly extract and reason over spatiotemporal dependencies, achieving a progressive representation learning. (III) Statistical Anomaly Decision: Finally, the reconstructed outputs are compared with ground truth to compute anomaly scores using a dynamic pruning strategy.

The proposed PSTG framework has a prediction-driven architecture that turns short-horizon forecasts and their deviations into anomaly evidence. An overview of the framework is illustrated in Figure 1, where *T* denotes the total sequence length, *L* is the context length, *F* represents the forecast horizon, and $n_{L}$ is the progressive composition depth.

PSTG is designed as a channel-agnostic and data-adaptive framework rather than a mission template-specific model. The multi-scale patch operator captures temporal behaviors at multiple horizons, the dynamic graph module learns couplings directly from observed telemetry interactions, and the statistical thresholding module calibrates alarm criteria from recent residual distributions. Because these components do not rely on explicit sensor identities or hand-crafted subsystem schematics, the same architecture is expected to transfer to other missions and subsystem groups with similar multivariate telemetry characteristics. This expectation is currently design-based and qualitative, and dedicated cross-mission validation will be addressed in future work.

The framework’s overall detection process acts on the full multivariate time series X and their consolidated predictions $\hat{X}$ to produce an anomaly score matrix S:

(1) $$\;S=\Phi \left(X,\;\hat{X}\right)\;.$$

The predictions $\hat{X}$ are generated by the core model, which maps a context window $X_{t-L+1:t}$ to an *F*-step forecast ${\hat{X}}_{t+1:t+F}$. This mapping is a progressive composition of three operators:

(2) $${\hat{X}}_{t+1:t+F}=T_{\Theta_{3}}\;\left(G_{\Theta_{2}^{\left(n_{L}\right)}}^{\left(n_{L}\right)}\circ G_{\Theta_{2}^{\left(n_{L}-1\right)}}^{\left(n_{L}-1\right)}\circ \cdots \circ G_{\Theta_{2}^{\left(1\right)}}^{\left(1\right)}\left(P_{\Theta_{1}}\left(X_{t-L+1:t}\right)\right)\right),$$

where $\Theta =\{\Theta_{1},{\left\{\Theta_{2}^{\left(l\right)}\right\}}_{l=1}^{n_{L}},\Theta_{3}\}$ denotes the set of learnable parameters associated with the multi-scale patching P, the stacked graph reasoning G, and the final forecast projection T, respectively.

The core operators of this framework, i.e., P, G, and Φ are defined as follows:

(1) **P: Multi-scale temporal patching.** P partitions raw telemetry data into a hierarchy of temporal patches and aggregates across scales, preserving fine-grained fluctuations and mission-level trends to yield a stable multi-resolution representation.
(2) **G: Progressive Spatiotemporal Graph reasoning.** G constructs a data-adaptive spatiotemporal dependency structure over channels and time, refining the latent representation via structure-guided attention. Executed in a stacked manner with depth $n_{L}$, it captures cross-channel couplings and long-range effects without assuming a fixed correlation pattern.
(3) Φ**: Statistical anomaly decision.** Φ converts forecast–signal discrepancies into anomaly evidence across channels and time and issues decisions under a data-calibrated criterion. Concrete choices (i.e., robust deviation, temporal stabilization, dynamic thresholding) are specified later.

### 3.2. Progressive Spatiotemporal Inference for Multi-Channel Telemetry

This section formalizes the core inference engine of PSTG, which progressively refines a latent representation of the input telemetry data through a deep, stacked architecture. The objective is to transform an initial multi-resolution embedding into a forecast-ready state by iteratively applying the spatiotemporal reasoning operator G.

Given the multi-channel telemetry sequence $X\in R^{C\times L}$, an initial latent representation, $Z_{\mathrm{fused}}$, is first generated by applying the multi-scale patch design operator:

(3) $$Z_{\mathrm{fused}}=P_{\Theta_{1}}\left(X\right).$$

$Z_{\mathrm{fused}}\in R^{C\times n}$ encapsulates hierarchical temporal features and serves as the initial hidden representation for the main reasoning stack, denoted as $H^{\left[0\right]}=Z_{\mathrm{fused}}$.

The progressive inference is defined as a sequence of transformations, in which each layer not only refines the representations produced by the preceding layer but also performs intra-layer spatiotemporal reasoning through the joint evolution of graph structure and attention weights via the operator G. Each layer possesses its own unique set of learnable parameters. The recursive update rule for the hidden states is formulated as:

(4) $$H^{\left[l\right]}=G_{\Theta_{2}^{\left(l\right)}}\left(H^{\left[l-1\right]}\right),\;\mathrm{for}\;l=1,\ldots ,n_{L}.$$

The final output of the stack, $H^{\left[n_{L}\right]}$, is the fully distilled, forecast-ready latent representation. This representation is then consumed by the prediction head: ${\hat{X}}_{\mathrm{future}}=T_{\Theta_{3}}\left(H^{\left[n_{L}\right]}\right).$

While the formulation above defines the transformation for a single context window, the complete predicted sequence, $\hat{X}$, is generated by deploying this entire inference pipeline in a sliding-window fashion across the full telemetry sequence, where each window produces a short-horizon forecast ${\hat{X}}_{\mathrm{future}}$ and only its first τ time steps are retained. These retained segments are then concatenated in temporal order to construct the final, continuous prediction $\hat{X}$. This global forecast $\hat{X}$ is then compared against the ground truth to identify anomalies, as detailed in the anomaly decision section.

#### 3.2.1. Multi-Scale Patch Design

Inspired by its success in natural language processing, patch embedding has recently emerged as a powerful paradigm for capturing local semantic information. Considering the large volume of telemetry data, we use a patch-based approach, dividing the data into smaller segments for analysis. This reduces the sequence length to be processed and lowers the overall computational load. Several existing methods rely on a uniform patch length. This single-scale approach, however, is inherently limited because a fixed patch size is ill suited to capturing both short-term fluctuations and long-term trends simultaneously.

This section provides the formal specification of the multi-scale temporal patching operator, $P_{\Theta_{1}}$. Conceptually, this operator is defined as the composition of three foundational transformations: a multi-scale partitioning function $\Pi_{P}\left(\cdot \right)$, a position-aware embedding function ${\mathrm{Embed}}_{\Theta_{\mathrm{emb}}}\left(\cdot \right)$, and a gated attention fusion function ${\mathrm{Fuse}}_{\Theta_{\mathrm{gate}}}\left(\cdot \right)$. The entire operator constitutes a mapping from the raw temporal domain to a structured, multi-resolution latent space, expressed as:

(5) $$P_{\Theta_{1}}={\mathrm{Fuse}}_{\Theta_{\mathrm{gate}}}\circ {\mathrm{Embed}}_{\Theta_{\mathrm{emb}}}\circ \Pi_{P}:R^{C\times L}\to R^{C\times N\times D}.$$

The learnable parameters of the operator are thus $\Theta_{1}=\Theta_{\mathrm{emb}}\cup \Theta_{\mathrm{gate}}$. Next, the concrete instantiation of each constituent transformation is specified.

The initial transformation, $\Pi_{P}\left(\cdot \right)$, discretizes the continuous input $X_{t-L+1:t}\in R^{C\times L}$ by partitioning it based on a set of *K* patch lengths $P=\{p_{1},p_{2},\ldots ,p_{K}\}$:

(6) $$\Pi_{P}\left(X_{t-L+1:t};P\right)={\left\{X_{p}^{\left(k\right)}\right\}}_{k=1}^{K}.$$

To ensure comparability, this function standardizes the output to a sequence of $N=\lfloor L/p_{\mathrm{main}}\rfloor$ patches for every scale, so that temporal patterns with different characteristic durations can be captured, by applying a sliding window with a calculated stride $h_{k}=\left\lfloor \frac{L-p_{k}}{N-1}\right\rfloor$. This yields a set of patches ${\left\{X_{p}^{\left(k\right)}\right\}}_{k=1}^{K}$.

The subsequent transformation, ${\mathrm{Embed}}_{\Theta_{\mathrm{emb}}}\left(\cdot \right)$, governed by the learnable embedding parameters $\Theta_{\mathrm{emb}}={\left\{\left(W_{k},b_{k}\right)\right\}}_{k=1}^{K}$, endows the partitioned data with semantic structure and temporal order:

(7) $${\mathrm{Embed}}_{\Theta_{\mathrm{emb}}}\left({\left\{X_{p}^{\left(k\right)}\right\}}_{k=1}^{K};W_{k},b_{k}\right)={\left\{Z_{k}\right\}}_{k=1}^{K}.$$

${\mathrm{Embed}}_{\Theta_{\mathrm{emb}}}\left(\cdot \right)$ is itself a combination of two functions. First, a scale-dedicated linear projection maps each patch to a *D*-dimensional vector. Second, to counteract the information loss from partitioning, a fixed sinusoidal positional prior is infused via summation. The complete embedding is defined as:

(8) $$z_{i,c,k}\;=\;\left(W_{k}\;x_{i,c}^{(k)⊤}+b_{k}\right)\;+\;p_{i},$$

where $W_{k}$ is the weight matrix and $b_{k}$ denotes the bias vector, and $p_{i}$ represents the fixed positional encoding vector for position *i*. The components of $p_{i}$ are defined by:

(9) $${\left(p_{i}\right)}_{j}=\left\{\begin{array}{cc} \sin(i/\theta^{2j/D}) & \mathrm{if}\;j\;\mathrm{is}\;\mathrm{even},\\ \cos(i/\theta^{(j-1)/D}) & \mathrm{if}\;j\;\mathrm{is}\;\mathrm{odd}. \end{array}\right.$$

Finally, the operator culminates in ${\mathrm{Fuse}}_{\Theta_{\mathrm{gate}}}$, governed by the learnable parameters $\Theta_{\mathrm{Gate}}=\left\{W_{\mathrm{gate}},b_{\mathrm{gate}}\right\}$, which adaptively aggregates the parallel, multi-scale representations into a single, unified representation. Its mapping is defined as:

(10) $${\mathrm{Fuse}}_{\Theta_{\mathrm{gate}}}\left({\left\{Z_{k}\right\}}_{k=1}^{K}\right)=Z_{\mathrm{fused}}.$$

After obtaining the set of embeddings $z_{i,c,1},z_{i,c,2},\ldots ,z_{i,c,K}$, ${\mathrm{Fuse}}_{\Theta_{\mathrm{gate}}}$ is used to fuse them into a single informative representation $z_{i,c}^{\mathrm{fused}}\in R^{D}$. Specifically, a gated attention mechanism is employed to compute the fused representation as:

(11) $$\begin{array}{cc} z_{i,c}^{\mathrm{fused}} & =\sum_{j=1}^{K}\alpha_{j}\cdot z_{i,c,j}, \end{array}$$

(12) $$\begin{array}{cc} \alpha_{j} & =\mathrm{Softmax}\;\left(\mathrm{Linear}\left([z_{i,c,1}∥\cdots ∥z_{i,c,K}]\right)\right), \end{array}$$

where the attention weights $\alpha_{j}$ are derived from a softmax function over a linear projection of the concatenated embeddings, allowing the model to learn the relative importance of each $z_{i,c,j}$ in the fusion process.

After fusing the temporal dependencies, we obtain the feature embeddings ${Z_{\mathrm{fused}}}^{,}\in R^{C\times N\times D}$, which are then reshaped into a node feature matrix $Z_{\mathrm{fused}}\in R^{n\times D}$. Here, each of the n=C×N rows represents a unique spatiotemporal node that will be processed by the subsequent graph reasoning module.

#### 3.2.2. Progressive Spatiotemporal Graph Modeling

The core of the proposed framework lies in the PSTG modeling module, which transforms the initial multi-resolution embedding, $Z_{\mathrm{fused}}$, into a forecast-ready state. This is achieved by iteratively applying a spatiotemporal reasoning operator, G, in a stacked architecture. The recursive update rule for the hidden states is given by:

(13) $$H^{\left[l\right]}=G_{\Theta_{2}^{\left(l\right)}}\left(H^{\left[l-1\right]}\right),\;\mathrm{for}\;l=1,\ldots ,n_{L},\;\mathrm{with}\;H^{\left[0\right]}=Z_{\mathrm{fused}}.$$

This section provides the formal specification for the generic operator $G_{\Theta_{2}^{\left(l\right)}}$ at any given layer *l*. Conceptually, the operator is decomposed into two primary transformations: a dynamic graph construction operator $G_{\mathrm{graph}}$, followed by a structure-guided graph attention operator $G_{\mathrm{attn}}$. The complete operator for a layer is thus expressed as:

(14) $$G_{\Theta_{2}^{\left(l\right)}}=G_{\mathrm{attn}}\circ G_{\mathrm{graph}}.$$

The learnable parameters are partitioned accordingly, and $\Theta_{2}^{\left(l\right)}=\Theta_{\mathrm{graph}}^{\left(l\right)}\cup \Theta_{\mathrm{attn}}^{\left(l\right)}$. The formal specifications for each constituent operator are provided in the following subsection.

##### Spatial–Temporal Graph Construction

The spatial–temporal graph construction approach formulates multivariate time series data as a dynamic graph, where the nodes represent spatial entities and the learned edges capture evolving spatial correlations [53]. To uncover the underlying relational structure among input variables without relying on a pre-defined static graph, a dynamic graph learning mechanism is applied to learn a sparse, weighted adjacency matrix directly from node features in an end-to-end manner.

Relying on a single graph structure can be insufficient to capture the complex, diverse nature of inter-variable dependencies. Therefore, the multi-head graph learner mechanism that allows the model to learn *H* distinct adjacency matrices in parallel from *H* different representation subspaces is adopted. Each head is specialized to capture a distinct pattern of the graph structure, such as dependencies at different time scales or of various types.

The operator’s mapping is defined as:

(15) $$G_{\mathrm{graph}}\left(H^{\left[l-1\right]};\Theta_{\mathrm{graph}}^{\left(l\right)}\right)={\left\{A_{\mathrm{final}}^{\left(h\right)}\right\}}_{h=1}^{H}.$$

The operator is parameterized by $\Theta_{\mathrm{graph}}^{\left(l\right)}=\left\{W_{1}^{\left(l\right)},W_{2}^{\left(l\right)}\right\},\mathrm{where}\;W_{1}^{\left(l\right)},W_{2}^{\left(l\right)}\in R^{\frac{D}{H}\times \frac{D}{H}}$. To maintain notational consistency with the preceding section, we continue to denote this input matrix as $Z:=H^{\left[l-1\right]}\in R^{n\times D}$, where $Z=Z_{\mathrm{fused}}$ for the initial layer (l=1).

To capture the spatial–temporal features of this node feature matrix, we first divided Z into different heads $Z^{\left(h\right)}\in R^{n\times \frac{D}{H}}$, where h∈{1,2,…,H}. To maintain parameter efficiency, two linear transformations with weights $W_{1},W_{2}\in R^{\frac{D}{H}\times \frac{D}{H}}$ are learned and shared across all heads. These transformations project the node features of each head into a relational space:

(16) $$E_{1}^{\left(h\right)}=Z^{\left(h\right)}W_{1},\;E_{2}^{\left(h\right)}=Z^{\left(h\right)}W_{2}.$$

The weighted adjacency matrix for each head is then computed via a dot product, followed by a Rectified Linear Unit (ReLU) activation:

(17) $$A_{\mathrm{dense}}^{\left(h\right)}=\mathrm{ReLU}\left(E_{1}^{\left(h\right)}{\left(E_{2}^{\left(h\right)}\right)}^{⊤}\right).$$

To enforce sparsity and retain only the most important connections, a top-*k* masking strategy based on a hyperparameter γ is adopted to obtain the final sparse adjacency matrix $A_{\mathrm{mask}}^{\left(h\right)}$ for each head.

To convert the raw edge weights into a normalized probability distribution, a softmax function is applied:

(18) $${\left(A_{\mathrm{norm}}^{\left(h\right)}\right)}_{i,j}=\frac{\exp\left({\left(A_{\mathrm{mask}}^{\left(h\right)}\right)}_{i,j}\right)}{\sum_{k=1}^{n}\exp\left({\left(A_{\mathrm{mask}}^{\left(h\right)}\right)}_{i,k}\right)}.$$

This step transforms the adjacency matrix into a row-stochastic matrix, where each row sums to one.

Finally, to prevent overfitting to the learned graph structure, we apply dropout with rate $p_{\mathrm{dropout}}$ directly to this normalized adjacency matrix during the training phase.

The final processed adjacency matrix ${\;A}_{\mathrm{final}}^{\left(h\right)}$ is then passed to the subsequent graph attention layer for information propagation.

##### Graph Attention Learning

Once constructed, the dynamic adjacency matrix ${\;A}_{\mathrm{final}}^{\left(h\right)}$ serves as the foundation for learning node representations. Our attention module uses a dynamic mechanism that computes attention weights based on pairwise interactions between a node and its neighbors, thereby enhancing the model’s expressive capacity. A key modification is introduced to the standard GATv2 architecture for more effective integration of the learned graph structure. Instead of applying a linear transformation to the concatenated query and key vectors, we directly use the learned adjacency matrix to modulate the attention scores. This design not only enhances computational efficiency by eliminating a linear layer but, and more importantly, directly injects the learned relational structure as a strong inductive bias into the attention mechanism.

The structure-guided graph attention operator $G_{\mathrm{attn}}$ is formally defined as:

(19) $$G_{\mathrm{attn}}\left(H^{\left[l-1\right]},{\left\{A_{\mathrm{final}}^{\left(h\right)}\right\}}_{h=1}^{H};\Theta_{\mathrm{attn}}^{\left(l\right)}\right)=H^{\left[l\right]},$$

where the learnable parameters for the layer are $\Theta_{\mathrm{attn}}^{\left(l\right)}=\left\{W_{Q}^{\left(l\right)},W_{K}^{\left(l\right)},W_{V}^{\left(l\right)},W_{O}^{\left(l\right)},w_{A}^{\left(l\right)}\right\}$.

Given the feature set Z, we first project it into a combined query Q, key K, and value V representation using three distinct linear layers. The resultant tensor undergoes reshaping and permutation to disentangle the representations for the *H* distinct attention heads. Specifically, Z is structured to yield *H* independent sets of $Q^{\left(h\right)}$,$K^{\left(h\right)}$,$V^{\left(h\right)}\in R^{n\times \frac{D}{H}}$, for h=1,…,H. To incorporate the explicit graph structure as a strong inductive bias, the learned attention scores are modulated by $A_{\mathrm{final}}^{\left(h\right)}$. The attention score from source node *j* to target node *i* is computed as:

(20) $$e_{i,j}^{\left(h\right)}={\left({\;A}_{\mathrm{final}}^{\left(h\right)}\right)}_{ij}\cdot \mathrm{LeakyReLU}\left(w_{A}\left[Q_{i}^{\left(h\right)}∥K_{j}^{\left(h\right)}\right]\right),$$

where $w_{A}\in R^{\frac{2D}{H}\times 1}$ is a shared linear projection applied across all heads.

These modulated scores are then normalized across all source nodes in the neighborhood of node *i*, using the softmax function to obtain the final attention coefficients $\alpha_{i,j}^{\left(h\right)}$:

(21) $$\alpha_{i,j}^{\left(h\right)}={\mathrm{softmax}}_{j}\left(e_{i,j}^{\left(h\right)}\right)=\frac{\exp\left(e_{i,j}^{\left(h\right)}\right)}{\sum_{k\in N_{i}}\exp\left(e_{i,k}^{\left(h\right)}\right)}.$$

To enhance regularization, dropout is applied to the attention coefficients. Subsequently, the message vector for node *i* within head *h*, denoted as $M_{i}^{\left(h\right)}$, is computed by aggregating the node features of its neighbors, weighted by the final attention coefficients:

(22) $$M_{i}^{\left(h\right)}=\sum_{j\in N_{i}}\alpha_{i,j}^{\left(h\right)}V_{j}^{\left(h\right)}.$$

The outputs from all *H* heads are then concatenated and passed through a final linear projection layer, $W_{O}\in R^{D\times D}$. This step yields the aggregated message representation $M\in R^{n\times D}$:

(23) $$M=\left(M^{\left(1\right)}∥M^{\left(2\right)}∥\ldots ∥M^{\left(H\right)}\right)W_{O}.$$

Finally, following the standard Transformer architecture, a residual connection is added to the input features, followed by layer normalization, to produce the layer’s final output, $Z_{\mathrm{out}}\in R^{n\times D}$:

(24) $$Z_{\mathrm{out}}=\mathrm{LayerNorm}\left(H^{\left[l-1\right]}+M\right).$$

#### 3.2.3. Loss Function and Optimization

To minimize the discrepancy between the multi-channel telemetry prediction and actual data, the learning criterion is formulated as a composite loss function L. This function is designed to capture the signal’s point-wise accuracy as well as its structural and dynamic properties, and is defined as:

(25) $$\begin{array}{cc} L(X_{\mathrm{future}},{\hat{X}}_{\mathrm{future}})\; & =\;\left(L_{\mathrm{MSE}}+\lambda_{1}L_{\mathrm{freq}}+\lambda_{2}L_{\mathrm{shape}}\right)\\ \; & =\;(∥X_{\mathrm{future}}-{\hat{X}}_{\mathrm{future}}{∥}_{F}^{2}+\\ \; & \;\lambda_{1}∥F\left(X_{\mathrm{future}}\right)-F\left({\hat{X}}_{\mathrm{future}}\right){∥}_{F}^{2}\\ \; & \;+\lambda_{2}∥\nabla_{t}X_{\mathrm{future}}-\nabla_{t}{\hat{X}}_{\mathrm{future}}{∥}_{F}^{2}), \end{array}$$

where ${∥\cdot ∥}_{F}$ is the Frobenius norm, F(·) represents the Discrete Fourier Transform (DFT) along the temporal axis, and $\nabla_{t}$ denotes the temporal gradient operator.The weight parameters $\lambda_{1}$ and $\lambda_{2}$ are critical for balancing point-wise reconstruction accuracy with spectral and structural properties. In this study, these hyperparameters were determined through a grid search on the validation set, ensuring that the model effectively captures both high-frequency fluctuations and long-term trends.

To optimize the model parameters, the aforementioned loss function is minimized using Stochastic Gradient Descent (SGD)-based methods. Specifically, the Adam optimizer [54] is adopted, which adaptively adjusts learning rates for each parameter and accelerates convergence. To further enhance convergence stability and generalization, the Cosine Annealing (CA) learning rate scheduler [55] is integrated, which gradually reduces the learning rate following a cosine decay schedule over the course of training. This scheduler is applied on top of the Adam optimizer to facilitate smoother model convergence by allowing for large initial learning rates and progressively finer updates as training proceeds.

### 3.3. Multi-Channel Telemetric Anomaly Detection

Following the prediction stage, which yields the output ${\hat{X}}_{\mathrm{future}}\in R^{C\times F}$, the final forecast sequence $\hat{X}$ is constructed using a recursive strategy where the number of retained points equals the window step size, τ. Specifically, only the first τ points from each prediction window are utilized. Once the complete forecast sequence is assembled, the anomaly detection phase begins. This process is designed to identify and score anomalous deviations by comparing the model’s predictions against the ground-truth telemetry data. The methodology adapts the robust, unsupervised techniques proposed by Kotowski et al. The overall procedure of the PSTG framework is summarized in Algorithm 1.

The application of the operator Φ is centered on the principle of non-parametric dynamic thresholding. Its core mechanism involves the determination of an optimal threshold, $\epsilon^{*}$, which is found by solving the following optimization problem over the raw residual sequence $r=|X-\hat{X}|$:

(26) $$\epsilon^{*}={argmax}_{\epsilon }\frac{\Delta \mu \left(r_{s}\right)/\mu \left(r_{s}\right)+\Delta \sigma \left(r_{s}\right)/\sigma \left(r_{s}\right)}{\left|r_{a}\right|+{\left|R_{\mathrm{seq}}\right|}^{2}},$$

where Δμ(r) and Δσ(r) respectively denote the decreases in the mean and standard deviation of the raw residuals after excluding values above the threshold ϵ; $r_{a}$ is the set of anomalous residuals exceeding ϵ; and $R_{\mathrm{seq}}$ represents the set of continuous sequences formed by those residuals.

Given the optimal threshold $\epsilon^{*}$ from the optimization step, the operator then assigns a severity score, *s*, to each detected anomalous sequence. This score quantifies the normalized magnitude of the deviation relative to the data-driven threshold:

(27) $$s^{\left(i\right)}=\frac{\max\left(r_{\mathrm{seq}}^{\left(i\right)}\right)-\epsilon^{*}}{\mu (r)+\sigma (r)}.$$

To enhance robustness, the operator’s application is extended with two refinements. First, to capture “silent failures” (i.e., anomalies manifesting as abrupt signal drops or inverted deviations), the entire optimization and scoring procedure is independently applied to a reflected residual sequence $r^{\mathrm{ref}}=2\mu \left(r\right)-r$. Second, to mitigate false alarms, a false-positive pruning strategy is employed, which assesses the percent decrease between the peaks of consecutively ranked anomalous sequences and reclassifies those below a predefined threshold $p_{\delta }$ as normal. This approach evaluates the percent decrease, $d^{\left(i\right)}$, between the peaks of consecutively ranked anomalous sequences, $r_{\max}^{\left(i-1\right)}$ and $r_{\max}^{\left(i\right)}$:

(28) $$d^{\left(i\right)}=\frac{r_{\max}^{\left(i-1\right)}-r_{\max}^{\left(i\right)}}{r_{\max}^{\left(i-1\right)}}.$$

If $d^{\left(i\right)}$ is found to be less than $p_{\delta }$, then the corresponding sequence and all subsequent, lower-ranked sequences are reclassified as normal.
**Algorithm 1** The complete PSTG algorithm  1:**Input:** Training dataset $D_{\mathrm{train}}=\left\{\left(X_{\mathrm{context}}^{\left(i\right)},\;X_{\mathrm{future}}^{\left(i\right)}\right)\right\}$; Telemetry sequence for detection $S_{\mathrm{test}}$; Setup of model hyperparameters for model training and prediction.  2:**Output:** Anomaly scores S for the sequence $S_{\mathrm{test}}$.    **Part 1: Parameter Learning via End-to-End Training**  3:Initialize parameters $\Theta =\{\Theta_{1},\;{\left\{\Theta_{2}^{\left(l\right)}\right\}}_{l=1}^{n_{L}},\;\Theta_{3}\}$.  4:Define the predictor$${\hat{X}}_{t+1:t+F}=T_{\Theta_{3}}\;\left(G_{\Theta_{2}^{\left(n_{L}\right)}}^{\left(n_{L}\right)}\circ G_{\Theta_{2}^{\left(n_{L}-1\right)}}^{\left(n_{L}-1\right)}\circ \cdots \circ G_{\Theta_{2}^{\left(1\right)}}^{\left(1\right)}\left(P_{\Theta_{1}}\left(X_{t-L+1:t}\right)\right)\right).$$  5:**for** epoch = 1 to *E* **do**  6:     **for** each batch $\left\{\left(X_{\mathrm{context}},\;X_{\mathrm{future}}\right)\right\}\subset D_{\mathrm{train}}$ **do**  7:          ${\hat{X}}_{\mathrm{future}}\leftarrow F_{\Theta }\left(X_{\mathrm{context}}\right)$  8:          $L\leftarrow L_{\mathrm{pred}}\left({\hat{X}}_{\mathrm{future}},\;X_{\mathrm{future}}\right)$.  9:          Update Θ.10:     **end for**11:**end for**12:$\Theta^{*}\leftarrow \Theta$.    **Part 2: Full Sequence Generation**13:$\hat{X}\leftarrow \left[\right]$.14:**for** t=L to length($S_{\mathrm{test}}$) - *F* step τ **do**15:     $X_{\mathrm{context}}\leftarrow S_{\mathrm{test}}\left[t-L+1:t\right]$.16:     ${\hat{X}}_{\mathrm{forecast}}\leftarrow F_{\Theta^{*}}\left(X_{\mathrm{context}}\right)$.17:     $\hat{X}\leftarrow \hat{X}\;\left|\right|\;{\hat{X}}_{\mathrm{forecast}}\left[1:\tau \right]$.18:**end for**    **Part 3: Anomaly Decision**19:$T_{pred}\leftarrow \mathrm{length}\left(\hat{X}\right)$.20:$X_{\mathrm{target}}\leftarrow S_{\mathrm{test}}\left[L+1:L+T_{pred}\right]$.21:$S\leftarrow \Phi (X_{\mathrm{target}},\;\hat{X})$.22:**return** S.

## 4. Experimental Results and Analysis

### 4.1. Description of Datasets

**Figure 2:**
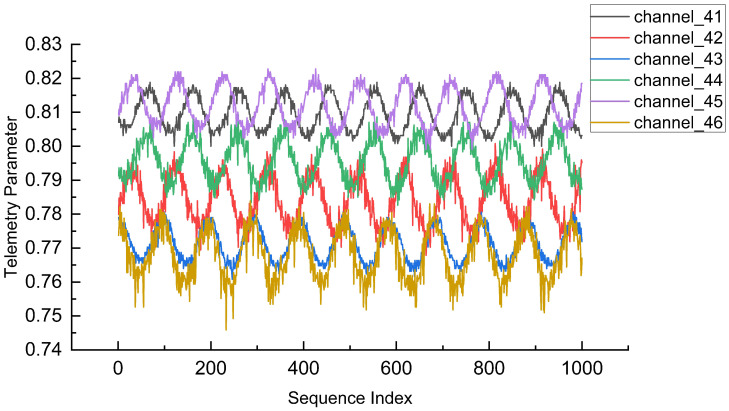
Partial illustration of the dataset.

Experiments were conducted using multi-channel telemetry data from real spacecraft, and the proposed algorithm was compared with eleven advanced methodologies to validate its effectiveness. Notably, the adopted European Space Agency (ESA) Anomalies Dataset (ESA-AD) conceals the telemetry channel names. This prevents the algorithm from leveraging domain-specific knowledge, emphasizing the use of a general data-driven approach rather than focusing on the complexity of specific tasks. The experiments were based on the lightweight subset from Mission 1 of the ESA-AD dataset, focusing on telemetry channels 41–46 of subsystem 5. This subset presented significant challenges due to the high number and complexity of anomalies. Given the advanced autonomous capabilities of modern spacecraft, individual telecommands exert less influence on telemetry behavior compared to earlier, non-autonomous systems. Consequently, the dataset used in this work excludes telecommand data. A representative segment of the Mission 1 lightweight subset is illustrated in Figure 2. The Y-axis can be omitted, since the channels have been normalized and vertically offset for visual clarity.

Both the training and test datasets consist of millions of telemetry data, with the final three months of the training set designated as the validation set. The validation and test sets contain only samples that occur after those in the training set to prevent data leakage from future time points. Anomalies are present in all three subsets—training, validation, and testing. This data-partitioning strategy maximizes the use of available data, reflecting a mature phase of the task where sufficient historical data enabled robust model training. Anomaly statistics across the three datasets are summarized in Table 1.

### 4.2. Experimental Details

The TimeEval [56] framework is employed, as modified by Krzysztof et al. [57], to implement the PSTG algorithm. The algorithm was first trained on the training set, during which contamination levels were calculated, thresholds were established, and standardization parameters were determined. It was then applied to the test set for online anomaly detection, operating without access to future samples from the test sequence.

In typical spacecraft mission operations, high-dimensional telemetry data are downlinked to ground stations for intensive health monitoring. Therefore, the PSTG model is positioned as a ground-based diagnostic tool, where the emphasis is placed on detection accuracy and interpretability rather than the extreme resource frugality required for on-orbit processing. Our model was implemented in PyTorch 2.6.0 under Python 3.9.0 and trained on a single NVIDIA GeForce RTX 4090 GPU. As outlined in Section 3.2.3, the AdamW optimizer was used in conjunction with a CA learning rate scheduler. The hyperparameters adopted in the primary experiment are summarized in Table 2.

For our method and all baseline approaches, the sliding window length *L* was set to 250 and the prediction window length *F* was set to 10, following the standard configurations established in Telemanom. From each prediction window, only the first time step (τ=1) was retained for further processing.

For anomaly detection, the size of the smoothing window is calculated by $W_{s}=p_{s}n_{s}B_{s}$, where $B_{s}$ is the test batch size, $n_{s}$ denotes a configurable base factor, and $p_{s}$ represents the tuning percentage.

The hyperparameters for the proposed model were carefully tuned and are detailed in Table 3.

### 4.3. Evaluation Metrics

Modified performance metrics from ESA-AD were adopted to better align conventional anomaly detection measures with practical spacecraft operational requirements. These metrics included the corrected event-wise F0.5-score and modified affiliation-based F0.5-score. Among these, the modified affiliation-based F0.5-score has a comparatively intricate formulation, so a dedicated description is provided in the following subsection. The computation of these metrics excluded the use of Point Adjustment (PA), a preprocessing protocol intended to refine anomaly predictions before evaluation. PA operates under the assumption that if any single point within an anomalous segment is correctly detected, then all points in that segment are treated as correctly identified. This protocol relaxes the detection burden on algorithms, though often leading to inflated performance scores [58]. The application of PA might allow randomly generated anomaly scores to surpass the performance of several recently proposed time series anomaly detection methods [59].

Operationally, Event-wise $F_{0.5}$ reflects whether anomaly events can be detected in time while controlling false alarms at the event level, which is critical for alarm triage in spacecraft operations. Affiliation-based $F_{0.5}$ evaluates temporal localization quality by measuring how well predicted anomaly intervals align with the corresponding ground-truth intervals in terms of boundary proximity and coverage completeness. Reporting both metrics therefore provides complementary evidence on alarm reliability and diagnosis usability.

Affiliation-based $F_{\beta }$-score is a time-domain metric that evaluates how closely and completely predicted anomaly intervals match each ground-truth interval by computing the average temporal distance within each exclusive affiliation zone:

(29) $$F_{\mathrm{aff},0.5}=1.25\cdot \frac{P_{\mathrm{aff}}\cdot R_{\mathrm{aff}}}{0.25\cdot P_{\mathrm{aff}}+R_{\mathrm{aff}}}.$$

Here,

$$\begin{array}{cc} P_{\mathrm{aff}}=\frac{1}{N}( & \sum_{j\in J}\left[\frac{1}{|\mathrm{pred}\cap I_{j}|}\int_{x\in \mathrm{pred}\cap I_{j}}{\bar{F}}_{\mathrm{precision}}\;\left(\min_{y\in {\mathrm{gt}}_{j}}\left|x-y\right|\right)\;dx\right]\\ \; & +(N-|J|)\cdot 0.5),\\ R_{\mathrm{aff}} & =\frac{1}{N}\sum_{j=1}^{N}\frac{1}{|{\mathrm{gt}}_{j}|}\int_{y\in {\mathrm{gt}}_{j}}{\bar{F}}_{\mathrm{recall}}\;\left(\min_{x\in \mathrm{pred}\cap I_{j}}\left|x-y\right|\right)\;dy, \end{array}$$

where $J=\{\;j|\mathrm{pred}\cap I_{j}\neq \emptyset \;\}$, *N* is the total number of ground-truth events, $I_{j}$ signifies the affiliation zone of ${\mathrm{gt}}_{j}$, $\bar{F}$ represents the survival function derived from uniform random sampling.

### 4.4. Baseline Methods

Baseline methods under comparison encompass a diverse set of approaches, ranging from classical techniques developed by NASA engineers [6] to competitive multivariate time-series modeling baselines. To broaden the comparison across modern modeling paradigms, high-performance time-series forecasting backbones commonly adopted in forecasting-based anomaly detection pipelines were incorporated, including the recently proposed deep spatiotemporal graph neural network TimeFilter [53]. Furthermore, several Transformer-based models that have emerged in the past two years were included, as they are widely recognized for achieving State-Of-The-Art (SOTA) performance in time-series forecasting, specifically iTransformer [39], PatchTST [40], and Crossformer [43]. Furthermore, lightweight MLP-Mixer-based alternatives such as DLinear [46] and TSMixer [41] are included to evaluate efficiency–accuracy trade-offs. Finally, the analysis considers methods that enhance deep neural network performance through frequency-domain or time-frequency decomposition techniques, such as FreTS [44], WPMixer [42] to assess robustness under diverse inductive biases. The modern forecasting backbones included in the benchmark are summarized in Table 4.

### 4.5. Anomaly Detection Results

The anomaly detection results of the baseline methods and PSTG are shown in Table 5. Evidently, no baseline method performed well across all five categories of metrics.

The accurate identification of anomalous events is paramount in spacecraft anomaly detection, with a primary focus on the event-wise F0.5 score, which places greater emphasis on precision to reduce false alarms. As shown in Table 5, PSTG achieves an event-wise F0.5 score of 0.917, significantly outperforming all competing baseline methods. In comparison, forecasting-based backbones such as PatchTST and iTransformer exhibit relatively strong recall but suffer from reduced precision, indicating a tendency to over-detect anomalies when applied to complex multivariate telemetry data. Simpler linear models (DLinear) and certain Transformer variants (Crossformer) fail to capture intricate cross-channel dependencies, resulting in substantially degraded event-wise performance.

Beyond event-level detection, anomaly detection systems must accurately localize the temporal extent of anomalous behaviors across multiple telemetry channels. The affiliation-based metrics explicitly evaluate the temporal alignment between detected anomalies and ground-truth intervals. PSTG attains an affiliation-based F0.5 score of 0.892 and consistently surpasses all baseline methods across affiliation-based precision, recall, and F0.5 metrics. While TimeFilter, as a graph-based baseline, demonstrates competitive temporal localization capability, its reliance on patch-level or static filtration limits its ability to progressively refine spatiotemporal dependencies.

Overall, the superior performance of PSTG across both event-wise and affiliation-based evaluations highlights the effectiveness of its progressive spatiotemporal graph reasoning mechanism for reliable spacecraft anomaly detection.

### 4.6. Discussion on Imbalanced Anomaly Patterns

The ESA-AD subset used in this work is naturally imbalanced, where normal samples dominate and anomaly events are relatively sparse. Under this setting, the $F_{0.5}$-oriented evaluation is intentionally adopted to emphasize precision and reduce operational false alarms. The dynamic thresholding strategy further mitigates spurious alarms by adapting to recent residual statistics; however, under extremely rare or weak anomaly patterns, recall degradation may still occur. These observations are reported as practical behavior under the current data regime rather than a complete robustness claim.

### 4.7. Explainability Analysis

**Figure 3:**
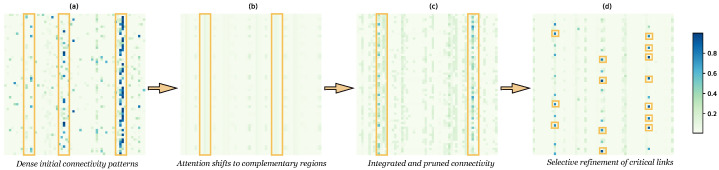
Visualization of adjacency and graph attention matrices in the two-layer ($n_{L}=2$) spatiotemporal graph neural network with multi-head attention (H=4). (**a**) Adjacency matrix after first layer. (**b**) Aggregated graph attention matrix after first layer. (**c**) Adjacency matrix after second layer. (**d**) Final graph attention matrix highlighting temporal, spatial, and spatiotemporal correlations.

As shown in Figure 3, relational patterns evolve progressively across network layers, illustrating how spatial, temporal, and spatiotemporal dependencies are incrementally refined. In the first layer, the adjacency matrix captures coarse spatial dependencies among nodes (Figure 3a), while the attention map displays diffuse and unstructured temporal interactions across time steps (Figure 3b). Following message passing in the second layer, the adjacency matrix becomes sparser and more structured (Figure 3c), indicating that the model selectively retains salient spatial connections while suppressing weaker or noisy ones. Finally, as shown in Figure 3d, the attention map exhibits concentrated regions of high intensity that correspond to correlated node activations across space and time, reflecting the emergence of stable spatiotemporal coupling patterns. This hierarchical refinement demonstrates that the proposed network adaptively enhances meaningful dependencies across spatial, temporal, and spatiotemporal dimensions, thereby improving both interpretability and representation stability.

To provide a rigorous mathematical foundation for the qualitative observations in Figure 3, we further quantify the evolutionary dynamics across the two stacked reasoning layers using Shannon Entropy (*H*). This metric evaluates the uncertainty and information distribution of the learned interaction components, defined as

(30) $$H\left(i\right)=-\sum_{j=1}^{N}p_{ij}\log\left(p_{ij}\right),$$

where N=60 represents the total number of spatiotemporal nodes (N=6 channels × 10 patches) and $p_{ij}$ denotes the interaction intensity between nodes. A higher *H* value indicates a more diffuse information integration, while a decreasing trend signifies the emergence of “reasoning determinism”. The quantitative results reveal distinct information-theoretic behaviors for the two interaction components: the entropy of the adjacency matrix exhibits a steady increase from Layer 1 (H≈2.94) to Layer 2 (H≈3.43), suggesting that as the reasoning depth increases, the model expands its receptive field, transitioning from initial local physical constraints to a more comprehensive, global representation of the spacecraft’s structural dependencies. Conversely, the attention mechanism shows a notable entropy reduction, dropping from H≈3.95 in Layer 1 to H≈3.54 in Layer 2, indicating that the model is actively “distilling” information by filtering out redundant telemetry noise and concentrating its representational capacity on a sparse subset of critical spatiotemporal interactions. This dual-process evolution—the expansion of structural context and the concentration of dynamic attention—mathematically confirms the PSTG model’s ability to balance holistic system monitoring with precise anomaly localization, ensuring the stability and interpretability of the learned coupling patterns for complex satellite health monitoring tasks.

**Figure 4:**
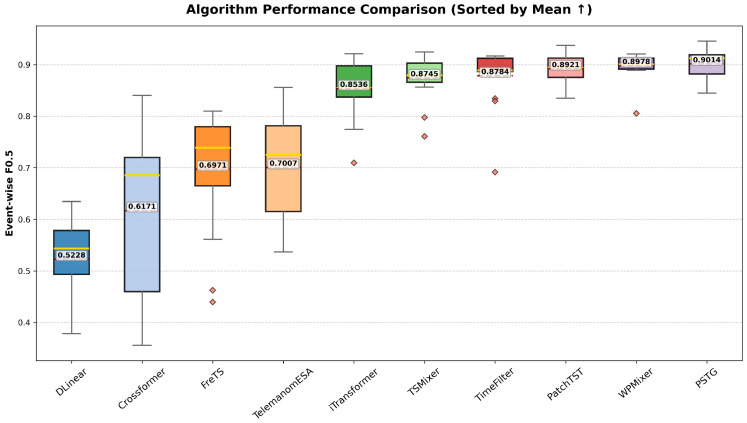
Statistical comparison of Event-wise F0.5 scores.

**Figure 5:**
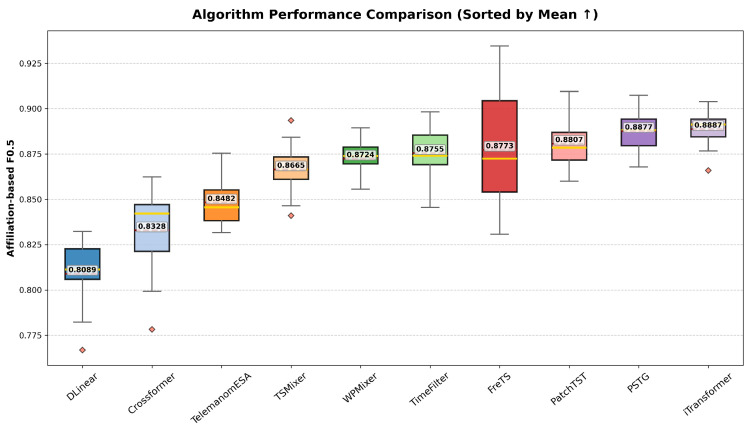
Statistical comparison of Affiliation-based F0.5 scores.

Across repeated runs, this entropy split (broader structural context with more focused attention) is consistent with the stronger and more stable Event-wise and Affiliation-based $F_{0.5}$ distributions reported in Figure 4 and Figure 5. This entropy–performance linkage is presented as an empirical consistency analysis for interpretability support, rather than a causal proof.

Figure 4 and Figure 5 summarize the statistical distributions of the F0.5 scores obtained from 15 independent runs across different baseline methods on the ESA dataset. Specifically, Figure 4 reports the Event-wise F0.5 results, while Figure 5 presents the affiliation-based F0.5 results. For each method, performance distributions are visualized using boxplots with per-run samples overlaid and sorted by the mean value, highlighting both the central tendency and variability of detection performance. This statistical comparison facilitates a direct assessment of robustness and stability of different approaches under repeated trials.

### 4.8. Ablation Study

To comprehensively evaluate component contributions, we report two complementary ablation settings.

**Figure 6:**
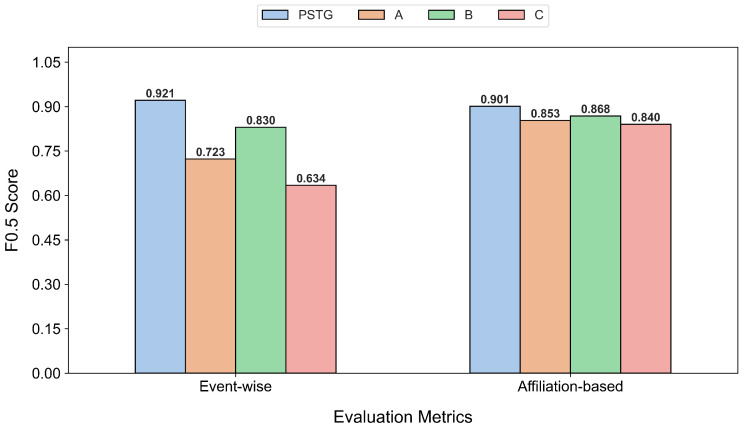
Performance comparison of PSTG vs. experiments A, B, and C.

**Backbone-level ablation (A/B/C+GCN).** We retain the original progressive degradation design: Experiment A replaces multi-scale patches with a single-scale variant, Experiment B further weakens graph construction by using single-head attention and removing top-*k* sparsification, and Experiment C additionally replaces attention-based aggregation with a GCN. As shown in Figure 6, performance decreases as these components are removed, confirming that multi-scale representation, sparse graph construction, and attention-based aggregation jointly support robust anomaly detection.

**Figure 7:**
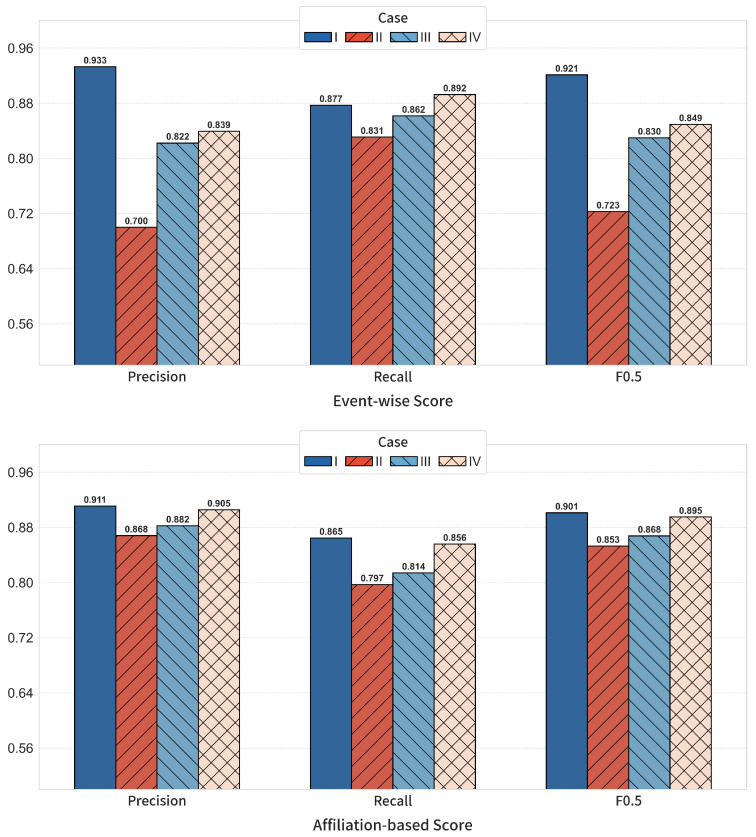
G-module-focused ablation results for four settings (I–IV). **Top**: event-wise precision, recall, and $F_{0.5}$. **Bottom**: affiliation-based precision, recall, and $F_{0.5}$.

**G-module-focused ablation (I–IV).** We further isolate the proposed attention architecture using four settings: Experiment I (full PSTG with M+A+G), Experiment II (w/o M), Experiment III (w/o A), and Experiment IV (w/o G, replacing the structure-guided attention with standard GATv2). As shown in Figure 7, Experiment I achieves the best overall scores. Removing M causes the largest drop in event-wise $F_{0.5}$ (0.921 → 0.723), and weakening A also reduces event-wise $F_{0.5}$ (0.921 → 0.830). The comparison between I and IV highlights the value of structure guidance: replacing the proposed module with GATv2 lowers event-wise $F_{0.5}$ from 0.921 to 0.849, with recall slightly increasing (0.877 → 0.892) but precision dropping markedly (0.933 → 0.839), indicating over-detection without explicit structural constraints.

## 5. Conclusions

In this work, the Progressive Spatiotemporal Graph (PSTG) framework was proposed to overcome the difficulty of modeling intricate, evolving dependencies in long-horizon spacecraft telemetry. By combining a multi-scale patch embedding strategy with a structure-guided graph attention mechanism, PSTG explicitly captures both global mission trends and local interaction dynamics across telemetry channels. Extensive experiments on an ESA real-world dataset show that PSTG outperforms eleven state-of-the-art baselines in almost all cases and, more importantly, bridges the gap between algorithmic prediction and operational trust by visualizing learned adjacency and attention matrices for actionable, interpretable alarms. Future work will extend this interpretability towards causal reasoning graphs and investigate lightweight model distillation for potential on-board deployment.

## Figures and Tables

**Table 1:** The lightweight subset dataset overview.

Mission 1–the Lightweight Subset	Train	Validation	Test
**Data points**	39,774,080	1,479,370	40,925,288
Duration (anonymized)	81 months	3 months	84 months
Annotated points [%]	1.74	1.23	1.81
**Annotated events**	**52**	**3**	**65**
Anomalies	22	2	29
Rare nominal events	26	1	36
Univariate/Multivariate	0/48	0/3	1/64
Global/Local	39/9	3/0	40/25
Point/Subsequence	1/47	2/1	9/56
**Distinct event classes**	**17**	**2**	**13**

**Table 2:** Setup of Hyperparameters for model training.

Parameters	Values
learning rate	$5\times 10^{-4}$
weight decay	$4\times 10^{-4}$
$T_{max}$	70
$eta_{min}$	0

**Table 3:** Setup of hyperparameters of the prediction model.

Parameters	Values
*P*	{25,50,125}
$p_{\mathrm{main}}$	25
*D*	512
*H*	4
$n_{L}$	2
γ	0.1
$p_{\mathrm{dropout}}$	0.1
$p_{\delta }$	0.21
$p_{s}$	0.05
$n_{s}$	30
$B_{s}$	70

**Table 4:** Modern time-series backbone baselines for spacecraft telemetry anomaly detection.

Category	Method	Key Insight/Architecture
**Modern TS** **Backbones**	DLinear [46]	A lightweight linear decomposition-based forecasting model that separates trend and seasonal components, serving as a strong linear baseline.
	iTransformer [39]	An inverted Transformer that captures multivariate correlations by embedding the entire time series.
	PatchTST [40]	A patch-based Transformer that preserves local semantic information and long-term dependencies.
	TSMixer/WPMixer [41,42]	High-performance MLP-based architectures that alternate between time and feature mixing.
	FreTS [44]	A frequency-domain MLP structure designed to capture periodic patterns in telemetry.
	Crossformer [43]	A Transformer variant designed to explicitly model cross-dimension and hierarchical dependencies.
	TimeFilter [53]	A spatiotemporal filtration approach that serves as a direct graph-based baseline.

**Table 5:** Performance comparison of PSTG and SOTA baseline methods on the ESA dataset.

Metric	Time Filter	iTrans Former	PatchTST	Cross Former	DLinear	TSMixer	FreTS	WPMixer	PSTG	
Event-wise	Precision	0.832	0.824	0.902	0.372	0.347	0.805	0.749	0.796	**0.932**
	Recall	0.846	**0.877**	0.862	0.815	0.846	0.769	0.831	0.846	0.862
	$F_{0.5}$	0.835	0.834	0.894	0.418	0.394	0.798	0.764	0.806	**0.917**
Affiliation-based	Precision	0.884	0.900	0.898	0.822	0.784	0.866	0.863	0.876	**0.905**
	Recall	0.814	**0.858**	0.837	0.768	0.705	0.775	0.797	0.782	0.844
	$F_{0.5}$	0.869	0.891	0.885	0.811	0.767	0.846	0.849	0.856	**0.892**

## Data Availability

The dataset (ESA-AD) supporting the findings of this study is openly available in Zenodo at https://zenodo.org/records/12528696 accessed on 6 April 2026; see the corresponding ESA-AD benchmark description in [57].
